# Does motivation and effort predict improvement on psychosocial functioning in schizophrenia (SZ)?

**DOI:** 10.1192/j.eurpsy.2025.2042

**Published:** 2025-08-26

**Authors:** X. Ansorena, E. Rosado, J. Chato, A. Zarzuela, A. Ballesteros, J. I. Arrarás, F. Gorriz, A. M. Sánchez-Torres, M. J. Cuesta

**Affiliations:** 1 Instituto de Investigación Sanitaria de Navarra (IDISNA); 2 Clínica de Rehabilitación de Salud Mental, Hospital Universitario de Navarra; 3 Departamento de Ciencias de la Salud, Universidad Pública de Navarra; 4 Servicio de Psiquiatría, Hospital Universitario de Navarra, Pamplona, Spain

## Abstract

**Introduction:**

Previous research suggests that motivational factors relate to psychosocial functioning in SZ, both concurrently (Tobe et al. Compr Psychiat 2016; 65 103-109) and at follow-up (Fervaha et al. Acta Psychiat Scand 2014; 130 290-299). Importantly, no study has examined the influence of baseline motivation on the *rate of change* in response to rehabilitation

**Objectives:**

1. To study the relationship between baseline measures of motivation/ effort with psychosocial functioning at follow-up
2. To examine if motivation/ effort predict individual change in psychosocial functioning

**Methods:**

**Participants**

Table 1 summarizes the sample characteristics

**Results:**

Figures 1 and 2 show individuals slopes for PSP and FAST, with a thick red line representing the average group slopes. For both PSP and FAST, models with only time as the independent variable and random intercepts indicated that time was a significant predictor (**PSP:** t=10.65, p<.0001; **FAST:** t =-6.13, p<.0001).

**
Baseline motivation/ effort → follow-up psychosocial functioning**

No significant correlations were found for neither PSP scores (**QLS:** ρ=-.018, S=2343.3, p=.93, **IMI:** P=.23, t=1.09, p=.28, **effort:** ρ=.001, S=2297.3, p=.99) nor FAST scores (**QLS:** ρ=-.16, S=2674.9, p=.45, **IMI:** P=-.02, t=-0.09, p=.92, **effort:** ρ=.07, S=2128, p=.72).

**Motivation → change in psychosocial functioning**

For PSP, the interaction model (Table 2) shows that the interaction of effort and timepoint significantly predicts PSP scores

**Table:** 

Variable	Frequency	Mean/ percentage	Standard deviation
Age	30	40.97	12.9
Gender	30		
Male	19	63%	
Female	11	37%	
Years of Education	24	11.42	3.06
Diagnosis	30		
… Schizophrenia	23	73%	
… Schizoaffective disorder	7	23%

**
Figure 1. Individual slopes for PSP scores**

**
Figure 2. Individual slopes for FAST scores**

**Image:**

**Figure:**
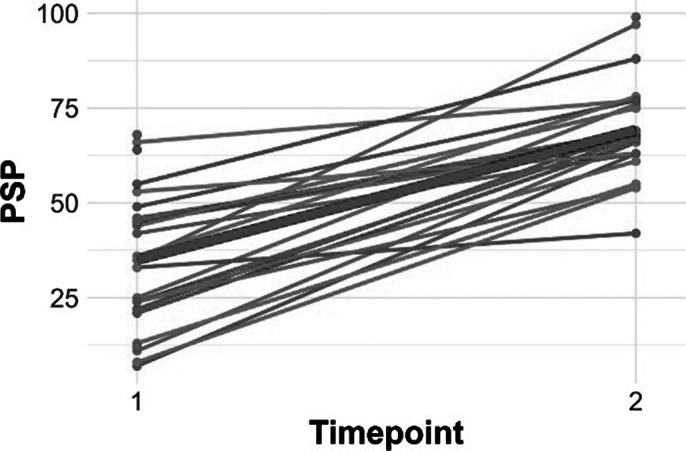

**Image 2:**

**Figure:**
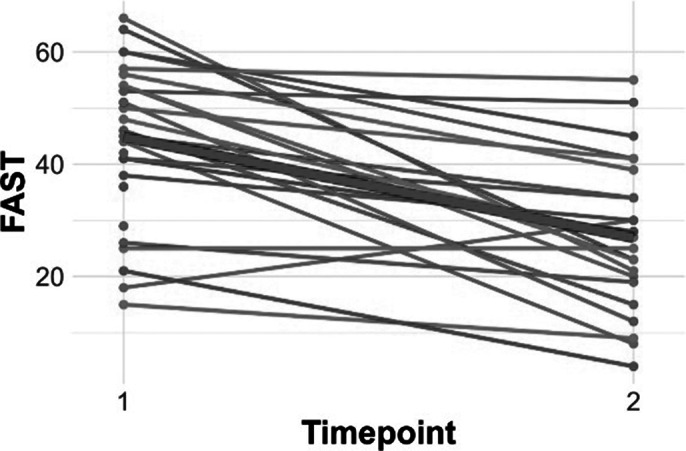

**Image 3:**

**Figure:**
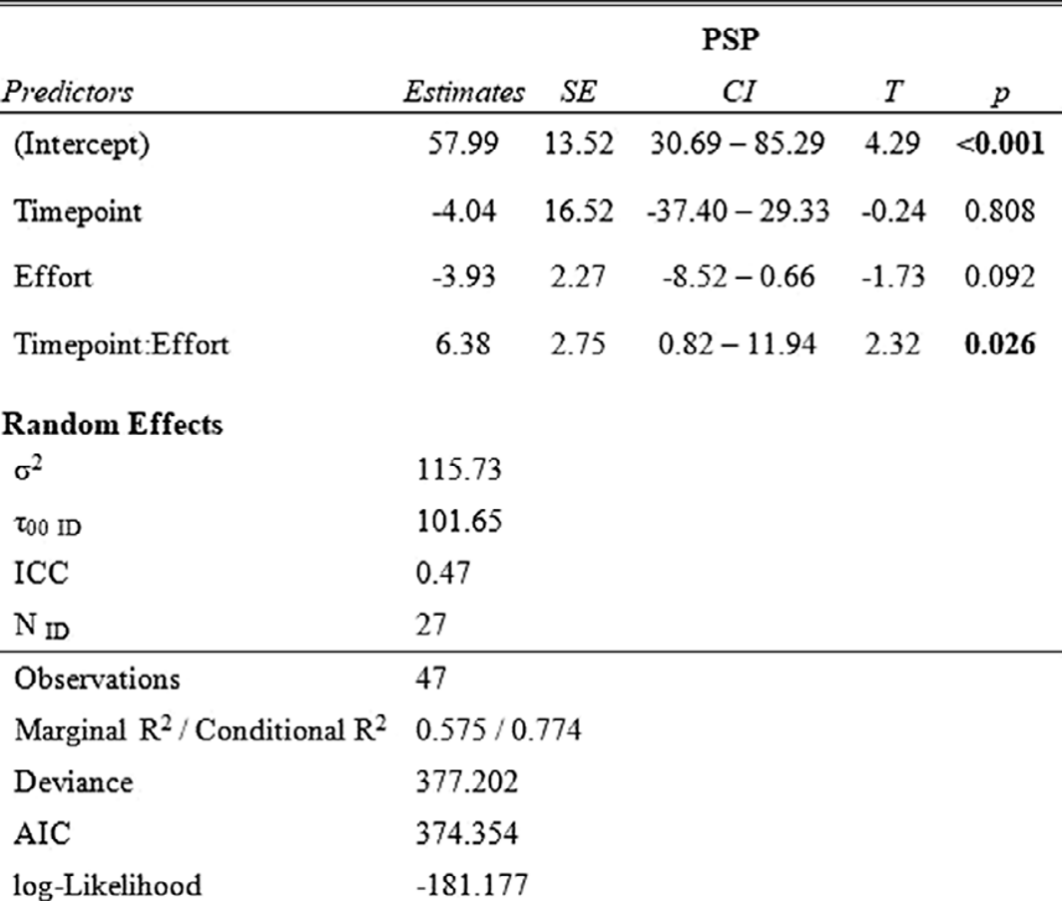

**Conclusions:**

Patients showed an improvement after rehabilitation. Effort can explain this trend. Finally, unlike previous studies, basal motivation did not predict follow-up psychosocial functioning

**Disclosure of Interest:**

None Declared

